# Fear and Trembling of Cruise Ship Employees: Psychological Effects of the COVID-19 Pandemic

**DOI:** 10.3390/ijerph17186741

**Published:** 2020-09-16

**Authors:** Aleksandar Radic, Michael Lück, Antonio Ariza-Montes, Heesup Han

**Affiliations:** 1 Independent Researcher, Gornji kono 8, 20000 Dubrovnik, Croatia; aleradic@gmail.com; 2 School of Hospitality & Tourism, Auckland University of Technology, Auckland 1010, New Zealand; michael.lueck@aut.ac.nz; 3 Department of Management, Universidad Loyola Andalucía, 14004 Córdoba, Spain; ariza@uloyola.es; 4 Faculty of Business Administration, Universidad Autónoma de Chile, Santiago 7500912, Chile; 5 College of Hospitality and Tourism Management, Sejong University, Seoul 05006, Korea

**Keywords:** coronavirus disease 2019 (COVID-19), psychological effects of the COVID-19 pandemic, qualitative methods, cruise ship

## Abstract

The current COVID-19 pandemic has evolved to unprecedented proportions. This research aimed to gain a deeper understanding of the psychological effects of the COVID-19 pandemic on cruise ship employees stuck at sea. Using an inductive qualitative approach, a synchronous online focus group was conducted with nine cruise ship employees who were stuck at sea during COVID-19 pandemic. The findings revealed that COVID-19 pandemic has managed to erase the feeling of joy from cruise ship employees who were stuck at sea while exposing weakness of cruise line companies such as poor human resource management leadership. Moreover, COVID-19 pandemic demonstrated that it is of paramount importance that cruise line companies create a comprehensive strategy in assisting their employees who are experiencing an anxiety disorder and depression. The managerial implications are outlined.

## 1. Introduction

Up until 2020, cruise tourism was the fastest-growing sector within the tourism industry [1]; however, on 14 March 2020, members of the Cruise Line International Association (CLIA) voluntarily suspended their cruise ship operation due to the COVID-19 pandemic, and on 12 April 2020 a No Sail Order issued by the Centers for Disease Control and Prevention (CDC) suspended all cruise operations until 30 September 2020 [2]. The Cruise Line International Association (2020) [3] outlines that 1.17 million jobs worldwide will be in danger due to the suspension of cruise operations caused by the COVID-19 pandemic. Furthermore, major cruise lines are experiencing enormous financial losses [4], and fears from the COVID-19 cruise tourism crisis have induced a devastating crash of major cruise lines stocks [5]. Thus, 122 new ocean-going ships that are on order until 2027 with a total value of 68.4 billion USD$ [6], are at risk due to the shrinking liquidity of the extremely fragile cruise line industry. Due to this poor liquidity and being on the verge of bankruptcy:

- Carnival Corporation has laid off 502 and furloughed 28 employees from their Miami-based office [7], laid off 450 from their UK-based office [8], and tapped into various financial agreements, raising 6.4 billion USD$ in liquidity [9];
- Royal Caribbean Cruises Ltd. has laid off or furloughed approximately 26% of their 5000 employees in the United States [10] and raised over $3.6 billion USD$ in liquidity [11];
- Norwegian Cruise Line Holdings Ltd. furloughed about 20% of its workforce including 4000 shore side employees and roughly 32,000 shipboard workers [12], and raised close to 3.5 billion USD$ in liquidity [13].

With suspended cruise ship operations and without revenue, cruise line companies are draining their funds at a fast rate. Balboa [14] predicts that from 15 May 2020, Carnival Corporation can sustain itself for 9 more months, Royal Caribbean Cruises Ltd for 11 more months and Norwegian Cruise Line Holdings Ltd for 18 more months. With such predictions, at least 250,000 crew members worldwide [15] are in danger of losing their jobs. 

Studies on well-being and life satisfaction of cruise ship employees are scarce; however, studies that are related to the psychological effects of being unemployed and isolated at sea due to the COVID-19 pandemic are to the authors’ best knowledge non-existent. Thus, the purpose of this research is to obtain a deeper understanding of the psychological effects of the COVID-19 pandemic on cruise ship employees stuck at sea. To accomplish this task, the following research question is addressed: “What are the psychological effects of the COVID-19 pandemic on cruise ship employees stuck at sea?”

This study is exploratory in nature and the present work addresses a major research gap. The aim of this research will be addressed by utilizing an inductive approach to data collection and an online synchronous focus group with cruise ship employees stuck at sea during the COVID-19 pandemic. Hence, to answer the research question and to fulfill the aim of this study, the specific objectives were to: (a) explore if cruise ship employees are experiencing certain worries, fears and sleep disturbances; (b) investigate how the lack of onboard social cohesion and lack of family and friends social support is affecting cruise ship employees; (c) assess components associated with perceived stress of cruise ship employees; and (d) evaluate the state of hope and sense of belonging of cruise ship employees. In light of the current COVID-19 pandemic, the results of this study provide valuable contributions to the theory of cruise tourism. 

## 2. Literature Review

### 2.1. Well-Being and Life Satisfaction of Cruise Ship Employees

Cruise tourism is a socio-economic system based on maritime transport with a sole purpose of creating tourism experiences founded on interaction between people, organizations, and geographical entities [16,17,18,19,20]. As such, cruise ship employees have a crucial role in delivering quality service [21] in a multi-sensory cruise experience [1]. Thus, the service quality and cruise experience are heavily dependent on the well-being and life satisfaction of cruise ship employees. Looking at cruise ship employees’ well-being, Radic et al. [22] argue that cruise ship employee well-being is a synthesis of happiness and pleasure. Similarly, the life satisfaction of cruise ship employees can be understood as an individual’s psychological aspects rooted in their hedonic satisfaction [23]. Considering the well-being and life satisfaction of cruise ship employees, Bardelle and Lashley [24] outlined how a large number of crew members are experiencing homesickness and sadness while working on a cruise ship. While investigating onboard experiences of cruise ship employees, Bolt and Lashley [24] found considerably stressful constraints that place pressure on cruise ship employees. Furthermore, Larsen at al. [25] found how life satisfaction of cruise ship employees was strongly influenced by respect, social atmosphere, and quality of food and living quarters. Hence, in a recent study on occupational health and safety of cruise ship employees, Radic [26] concluded how work-related injuries have profound negative effects on the well-being of cruise ship employees, creating a perception of unattractive and unfavourable working conditions among crew members. Interestingly, cruise ship employees who resigned from cruise line companies to start a new beginning with a land-based job, exhibit a certain degree of nostalgia with a romanticized feeling about well-being and life satisfaction while being onboard [27]. However, while being onboard, cruise ship employees are exposed to prolonged harsh working conditions in the form of constant time pressure and heavy workload coupled with the everlasting uncertainty about their next contract assignment [28]. Moreover, unfavorable working conditions combined with the inability of psychological detachment from the work creates a negative impact on cruise ship employees’ well-being [29]. Thus, Cruise Industry News [21] argues how poor well-being of cruise ship employees can affect their mental health, which leads to high employee turnover, absenteeism, and increased expenses due to health care costs. Hence, it appears that cruise ship employees are trapped in what Moore [30] calls “misery machines”. Peculiar work and life conditions of cruise ship employees affect their well-being to the point of the alarming rise of suicide rates in recent years [31,32]. The Shipowners’ Club [33] briefly outlines that cruise ship employees’ mental and physical health and relationships at home and onboard were evidently affected by the COVID-19 pandemic. In summary, although aforementioned studies have provided a glimpse of cruise ship employees’ well-being and life satisfaction, currently there exists a research gap on the psychological effects of a pandemic, in particular the COVID-19 pandemic on cruise ship employees stuck at sea. 

### 2.2. Ethical Statement

Because of the observational nature of the study, and in the absence of any involvement of therapeutic medication, no formal approval of the Institutional Review Board of the local Ethics Committee was required. Nonetheless, all subjects were informed about the study and participation was fully on voluntary basis. The study was conducted in accordance with the Helsinki Declaration.

## 3. Methodology

### 3.1. Overview and Qualitative Procedure

This research is explorative as it analyzes new and unprecedented areas of the COVID-19 pandemic. The goal of this study is to explore the psychological effects of the COVID-19 pandemic on the cruise ship workforce stuck at sea. Taking into consideration that the COVID-19 pandemic has led to a health and epidemiological type of crisis in cruise tourism, this novel situation needs to be explored in-depth, and qualitative research with an interpretivist paradigm was adopted. Interpretivism argues that social phenomena should be studied from the perspective of involved social actors [34]. Communication and social relationships are comprised of diverse expositions and individual responses, and, as such, interpretivist-qualitative lenses provide a meticulous understanding of the connection between implication and action [35]. Furthermore, the interpretivist paradigm creates opportunities for accessing diverse expressions and points of view since this paradigm seeks to raise the voices of the community in the appraisal process [36].

### 3.2. Research Design and Data Analysis

This study took an inductive approach and a qualitative method where an online synchronous focus group was conducted. The synchronous online focus group is a peculiar situation where the moderator and somewhere between four to nine participants are in an online chat room where everyone simultaneously types comments that are visible to all group members [37]. 

Possible participants were invited to take part in the research via the Crew Center Facebook group that connects cruise ship employees. Participants for this study were chosen using a convenience sampling method. To participate in the synchronous online focus group, the participants had to meet the following criteria: (1) being stuck at sea on a cruise ship during the interview; (2) being on a cruise ship since 14 March 2020, the day when members of the CLIA voluntarily suspended their cruise ship operation; (3) being without a contract and not being paid while being stuck at sea; and (4) not knowing their repatriation date. Data were collected from nine cruise ship workers employed by four major cruise lines and who were on nine different cruise ships during the COVID-19 pandemic. These cruise ship employees are members of various onboard departments. Given the aim of examining the psychological effects of the COVID-19 pandemic on cruise ship workforce stuck at sea, cross-sectional research was conducted. The major strength of a cross-sectional design is convenience, as such studies are quick to complete and relatively inexpensive [38], and they provide a picture of a situation related to a particular population within a specific time [39]. In this study, a synchronous online focus group was conducted via the well-known text and voice messaging cross-platform WhatsApp. This option ensured participants’ anonymity since they are not visible to the group and the recent popularity of chat emoticons buffered the disadvantage of not having visual cues [35]. The study was conducted on 22 May 2020 and the online synchronous focus group lasted 68 min. One of the authors who works on the cruise ship and was stuck at sea since 14 March 2020 when members of the CLIA voluntarily suspended their cruise ship operation, acted as a moderator. At the end of the discussion, the moderator downloaded the entire script. Written data were coded using open and axial coding techniques grounded in procedures outlined by Strauss and Corbin [40]. 

Online synchronous focus groups are not without shortcomings, however, rigor was achieved by following Higginbottom’s [41] recommendations, carefully recruiting participants based on their experience and knowledge. Validity was enhanced by facilitating a lavish data set as recommended by Morse [42]. Reliability was ensured by following Richard et al.’s [43] recommendations with a skilled and experienced researcher (one of the authors) acting as moderator, which was instrumental in overcoming the disadvantages of lack of non-verbal cues, minimizing disturbing conduct of some of participants, and minimizing various errors and biases. 

In summary, an inductive approach and a qualitative method with an online synchronous focus group are commonly used for topics that are not well understood so the new insights could be discovered. This study aims to explore the psychological effects of the COVID-19 pandemic on cruise ship employees stuck at sea. Its explorative nature made online a synchronous focus group a suitable method for this study. Furthermore, as an exploratory study on the psychological effects of the COVID-19 pandemic, the portrayal of the sample as part of a specific entity is not a considerable worry as the method aims to investigate the aspects and provide a structure instead of variables estimation and their description. 

## 4. Psychological Effects of the COVID-19 Pandemic on Cruise Ship Employees Stuck at Sea

### 4.1. Demographic Profile of Participants

Respondents from the synchronous online focus group were cruise ship employees who were on board since 14 March 2020; they were without a contract and not being paid, and they did not know their repatriation date. The respondents’ age ranged from 26 to 43 years; five respondents were from Asia, followed by three respondents from Europe, and one respondent from South America. The sample was evenly distributed in terms of gender, with five females and four males. Looking at the participants’ working departments, the sample had seven respondents from the hotel department, one participant from the marine and technical department, and one participant from the entertainment department (Table 1).

### 4.2. The Anxiety and Its Effects on Cruise Ship Employees

The Maritime Labour Convention 2006 [44] “seafarers’ bill of rights” was extended to cruise ship seafarers in 2013, and under the “seafarers’ bill of rights”, cruise line companies are obliged to provide for their crew members repatriation at the end of their contract. However, on 12 April 2020, due to the COVID-19 pandemic, the CDC issued a No Sail Order, which has prohibited cruise line companies to use any form of commercial transportation for crew member repatriation purposes [2]. The CDC’s No Sail Order in combination with poor liquidity of cruise line companies due to the COVID-19 cruise tourism crisis has created an unprecedented event leaving 100,000 cruise ship employees stuck at sea for months without any certainty when they will be repatriated to their homes [45]. Consequently, like Song and Li [46] argue, the uncertainty of what future might hold can lead to anxiety. During the discussion, all participants have outlined that they are experiencing certain worries while being stuck at sea.

*My contract was until July 2020. The company unilaterally stopped it on 30 March and since then (today is 22 May) they are holding me onboard without pay. My family needs financial support and I am unable to provide them financial support because my company doesn’t want to pay for the charter flight, and the CDC is not allowing the company to send me home with a commercial flight.*

(Cruise ship employee No. 4)

Cruise ship employees’ worries are related to not being able to provide financial support to their families, not being able to see their family and friends, and ultimately they are left with a feeling that they do not have any control of their life. They are experiencing negative economic and social effects of being unemployed and isolated at sea due to the COVID-19 pandemic. Thus, the aforementioned conditions can lead to anxiety, where House and Stark [47] define anxiety as a comprehensive adaptive reaction when the individual is facing an unknown danger. Moreover, due to the COVID-19 pandemic, government-issued isolations will have an enormous negative effect on mental health for many, especially on those individuals who are peripheral members of the society, because they are more likely to face financial deprivation and lower quality of life [48]. Although cruise ship employees come from various countries around the globe, the majority of them are from undeveloped or developing countries [49], thus, cruise ship employees’ mental health will be most likely affected by the COVID-19 pandemic.

The anxiety of cruise ship employees stuck at sea is growing as they fear an uncertain future and an economic mega-crisis that lies ahead. Increased anxiety and fear-associated traits within human behavior are related to the fearful stimuli and increased activity in the amygdala. It is well documented that fear spreads extremely fast and that no one is immune to fear [50]. During the discussion, all respondents outlined that their biggest fears were related to not being able to see their family, not being able to go home, not getting paid, the uncertainty of what future holds, and knowledge of forthcoming financial deprivation.

*I fear that my son won’t recognize me when I come home. I am already 9 months onboard. I have to wear a mask while I talk to my son, and he always asks me to remove my mask because he can’t see me. I can’t hold off my tears when he tells me this.*

(Cruise ship employee No. 2)

Seafaring is an occupation that carries hardship [51], and the COVID-19 cruise tourism crisis illustrated that cruise ship employees feel afraid, lonely, unprotected, and financially enslaved. Living in fear is what makes one “being a slave” [52] and since cruise ship employees are low paid, mobile, and under “Maltese contract,” “Cyprus contract,” or “Swiss contract”, they fit well in what Mann [53] describes as wage slaves. Nevertheless, cruise ship employees come onboard with hopes that their work efforts will in return provide them and/or their family considerable financial benefits [49]. Furthermore, the COVID-19 cruise tourism crisis has shattered, both in ethical and aesthetic nature, cruise ship employees’ dreams and ideals of a brighter future. However, Kierkegaard [54] argues how such destruction leads only to the remolding of dreams and ideals on new grounds.

Sleep is a process that incorporates neurobiological, neurochemical, and psychological systems [55]. Moreover, an optimal period of quality sleep plays an important function as a safeguard for individuals’ mental health and everyday performance [56]. Abysmal sleep quality can lead to quite a few psychological disorders including depression, anxiety, and paranoia [57]. Thus, sleep disturbances are one of the symptoms of anxiety [58]. Gillespie [59] discusses how isolation and coronavirus anxiety lead to insomnia. During the discussion with the participants, every single one of the participants described in an individual way how their quality of sleep was poor.

*I am constantly tired although I am not working. With too much pressure on my shoulders, too much free time to think about all the possible things that can go wrong, locked inside my cabin, when night comes I can’t fall asleep. I force myself to sleep, only to wake up every 2–3 h. looking at my watch asking myself when will this agony come to an end.*

(Cruise ship employee No. 9)

A clear sign of cruise ship employees’ growing anxiety is their sleep pattern. As days onboard become weeks, and weeks become months, cruise ship employees experience insomnia and when they fall asleep, nightmares wake them in distress. Sleep disturbances are a common factor in anxiety disorders where complaints related to insomnia and even nightmares are fundamental in defining generalized anxiety disorder and even posttraumatic stress disorder [60]. Worries, fears, uncertainty, isolation in small cabins, lack of opportunity to share one’s concerns, and loud noise from maintenance were just some of the factors that have led to sleep disturbance and anxiety of cruise ship employees. Moreover, due to the CDC’s No Sail Order [2], cruise line companies are failing to meet obligations related to cruise ship employees’ living conditions, recreational areas and amenities set in The Maritime Labour Convention 2006 [44] “seafarers’ bill of rights”. 

### 4.3. Depression and Its Effects on Cruise Ship Employees

Depression is a profound medical illness that negatively affects how an individual feels, thinks, and acts, which ultimately leads to sadness and/or a deprivation of delight in previously pleasurable activities [61]. Seafarers are susceptible to diverse mental health disorders including depression [62]. Bearing in mind that social isolation is a robust contributor to depression [63], cruise ship employees that are stuck at sea for months [45] due to the COVID-19 pandemic are at risk to aggravate depressive symptoms. During the discussion, all respondents clearly outlined some traits of depression.

*This hopelessness, confusion, sadness, and longing. Some of us have finished our contracts two months ago and since then we are not paid. There are people on board who are here for more than 7 months. It’s very hard for all of us and none of psychologist and none of that nonsense talk will help us. Just look at the people who have committed suicide in the last 10 days. I think there were 4 or 5 suicides by crew members who were stuck at sea. This is terrible. People are on the edge and most of the people are broken beyond repair.*

(Cruise ship employee No. 7)

Prolonged isolation, despair, deterioration of well-being, and impossibility to return to their homes so they can be reunited with their loved ones have seriously affected how cruise ship employees feel, think, and act. Although evidence on seafarers’ depression and suicide rates are scarce and fragmented [64], while being stranded at sea due to the COVID-19 pandemic, four crew members have died under unclear circumstances and not related to the COVID-19 virus [65]. Cruise ship employees who embark on the cruise ship in pursuit of a brighter future found themselves in what Kulzer et al. [66] describe as a world where fear survives. Cruise ship employees are facing a transformational experience that Rogell et al. [67] pronounce as the journey of a lifetime.

Humans are social beings that are constructing the hierarchical structure of society to obtain and maintain resources [68]. To comply with The Maritime Labour Convention 2006 [44] “seafarers’ bill of rights”, cruise line companies are in obligation to provide recreational facilities and amenities for socializing purposes of their crew members. However, the CDC’s No Sail Order [2] has specifically forbidden the usage of recreational facilities and amenities for socializing purposes. Consequently, the CDC’s No Sail Order [2] has created both objective and subjective social isolation. Subjective social isolation from both family and friends is associated with higher depressive symptoms [69]. Participants have made a clear statement that it is impossible to socialize due to the CDC’s No Sail Order [2].

*By some law, we should have recreational space and space for socializing like crew bar or similar space. The CDC forbids the usage of crew gym, crew bar, or anything where we can group for socializing. I come from a society where we live in small and large groups caring for each other. This is also how we behave while we are on the ship. Now we can’t do that, so I fell very lonely, sad, and depressed.*

(Cruise ship employee No. 3)

Cruise ship employees socialize with one another in specially designated bars, while they are at the gym, on the rare occasion when they go ashore, and some of the crew members even engage in casual intimacy [49]. However, while being stuck at sea due to the COVID-19 pandemic, cruise ship employees feel alone, isolated, depressed, and detached because they are unable to enjoy onboard socialization due to the CDC’s No Sail Order [2]. The liminality of the cruise ship and rigid managerial hierarchical structure erase a clear line between private life and workplace of crew members [70], thus, while being stranded at sea, cruise ship employees have lost all points of reference except daytime and nighttime. 

Human beings are social animals [71] in need of relatedness to friends and family. However, cruise ship employees understand that to the cruise line companies they are nothing more than a number on their identification card, thus, for the cruise ship employees’ perceived social support and relatedness to friends and family needs satisfaction is of paramount importance [49]. Furthermore, the quality of family interactions is of utmost importance for understanding the development process of depressive symptoms in adolescents [72]. Depressive symptoms decrease significantly with those individuals who enjoy strong family and spousal support [73]. During the discussion, participants used every opportunity to stress how much they are missing their friends and family at home.

*Yes, I miss my family and friends from home very much! The internet is very, very slow and sometimes we are without a connection for 5 to 6 times per day. The company refuses to increase the speed of the Internet. So if we buy an internet package then the internet works fast and the connection is not interrupted. I wonder how is that possible? I can barely get in touch with my lovely mom to cheer me up. I can’t use video calls, I can’t send/receive pictures and definitely, I can’t send video. I cannot see my family. I think some of us have purchased an Internet plan and we are not under contracts and not getting paid more than 60 days.*

(Cruise ship employee No. 8)

Under normal circumstances cruise ship employees leave their family and friends at home and the mental pressure of such decision weighs heavy on them. Crew members cope with such hard decisions by psychologically preparing themselves that their sacrifice will provide them and/or their families with considerable financial benefits [49]. However, in a case when cruise ship employees are stuck at sea due to the COVID-19 pandemic, where the majority of them are not getting paid or are paid a minimal salary, the uncertainty of when they will go home, if they will find another job and what would be a new reality when they eventually go home, is crushing them. 

### 4.4. The Stress and Its Effects on Cruise Ship Employees

Human functional brain networks is a system that organizes various assignments such as planning, anticipating, analyzing (executive control network), reflecting on previous experience (default mode network), determining the importance of the current environment (salience network), and focusing attention to the issue at hand (ventral attentional networks) [74]. On 11 April 2020, cruise ship seafarers went to sleep with hopes that they will soon go to their homes and loved ones, however, they woke up on 12 April 2020 in a completely different world where the CDC’s No Sail Order [2] was not allowing them to use any form of commercial transportation for repatriation purposes. Thus, since that point in time, cruise ship employees have been under chronic stress, due to situations where salience brain network activity (scanning the environment for threat) has taken control and executive control brain network (analyzing current conditions) has been deactivated. Fear is a mind killer [75], and being afraid of something suppresses the ability to think straight [74]. As a consequence, Sep et al. [76] conclude how extrapolation of fear is a frequent indication of anxiety and trauma-related disorders. During the discussion, participants clearly outlined how they feel agitated due to the uncertainty of what the future holds.

*I am in no control of my own life. I am afraid of what tomorrow will bring. I feel paralyzed and I can’t think straight anymore. On top of that I live in a small dirty cabin, every day I experience poor crew office service, I don’t feel respected by anyone, I can’t eat at the buffet when I feel hungry, I can’t buy alcoholic drinks as much as I want to, I don’t have decent internet service. When I call to crew office to ask about things that are broken in my cabin – they hang up the phone on me. People are getting rude. I just want my life back!!! I’m fed up, fed up, and fed up!*

(Cruise ship employee No. 5)

The COVID-19 cruise tourism crisis managed to erase the feeling of joy from cruise ship employees, thus, it does not come as a surprise when they speak in an almost passive participant voice about their relentless complaints against diminished control of their own life. It appears as though fear of what might come has spread across the cruise ships. Thus, melancholy prevents cruise ship employees from feeling any positive emotions. As the uncertainty of future events keeps on suffocating cruise ship employees, they grow agitated, confused, and depressed. 

For inexperienced seafarers, cruise ships appear as delightful ocean-floating hotels with never-ending entertainment, and a wonderful way to obtain monetary gain while being able to visit exotic places. However, cruise ship employees work and live on board for a prolonged period of time, and during their stay on board, they are often exposed to various stressful events that affect their life satisfaction [77]. In light of the COVID-19 pandemic, cruise ship employees that are stuck at sea are experiencing particularly high levels of stress that may develop mental health disorders such as anxiety and depression. During the discussion, all participants but one expressed how they feel stressed and nervous.

*I feel stressed and furious. If the President of the IMO, CDC, or whatever had a kid or spouse stuck on a ship would they not do everything to get them home? It’s common sense to allow crew members to go to their homes. Did you know that 5 crew members committed suicide in the last 10 days? Do you know why they did it? Because they couldn’t cope with stress, so what do you think will happen if this drags for another two months or until 24 July 2020 when the No Sail Order comes to an end?! Governments around the world need to step up and help their citizens who are crew members, and the CDC needs to come forward towards an agreement with an international organization and various governments.*

(Cruise ship employee No. 4)

While being stuck at sea, cruise ship employees are caught in no man’s land between: (1) cruise companies on verge of bankruptcy that are trying to consolidate their liquidity; (2) a distant CDC administration who are inhumanly insisting on noncommercial transportation for crew members’ repatriation purposes; and (3) quite a few government bureaucracies who are not allowing their crew members to come back home. Due to the fear of uncertainty, stress builds up and it appears that on the high seas no one can hear crew members’ silent screams of existential despair. Lastly, as reconstructions in fear neurocircuitry influence anxiety disorder, chronic stress manifestation reorganizes fear neurocircuitry, triggering structural degeneration in the prefrontal cortex and hippocampus, thereby restricting dominance over the stress response [78].

The COVID-19 pandemic changed the world, and each human being has changed in their own way. When it comes to cruise ship employees, the COVID-19 cruise tourism crisis showed that major cruise line companies do not have a contingency plan in case of a health and epidemiological type of crisis and that many governments do not have coherent leadership. Consequently, cruise ship employees found themselves alone in a chaotic situation. During the discussion, the majority of participants shared the opinion that they cannot cope with all the things that are happening in the world during the COVID-19 pandemic.

*Every question we asked we get an answer “We don’t know” or “We are not sure”. We are really worried about our future and our mental health because this is a situation without a solution. We are not getting paid, we can’t buy drinks, the gym is closed, and there are no activities that would relax us here onboard. When you want to buy morning coffee you need to wait in line for 1 h, because the ship is understaffed. Of course all of us are very stressed in this situation. Today security called us in cabins to ask for the names of people who were protesting yesterday. It is serious retaliation and this is against human rights. It feels like none of us have a right to say our opinion. We just want the world to hear us because all of us just want to go home to be with our families.*

(Cruise ship employee No. 1)

Cruise ship employees who are stuck at sea have found themselves in a peculiar situation that they cannot change because they depend on multilateral dialog and agreements between cruise line companies, the CDC, airlines, and various governments. As uncertainty crawls in every pore of cruise ships, causing waves of stress, depression, anxiety, and panic, cruise ship employees are in need to create a common sense from what appears to be a hopeless situation. The COVID-19 pandemic is having an overwhelming effect on all life aspects of cruise ship employees, including mental and physical health. Thus, as Frankl [79] concludes, when a person finds themselves in a situation that they cannot change, the only thing the person can do is to embrace the opportunity to change themselves. 

### 4.5. A New Hope for Cruise Ship Employees

While cruise line companies are dealing with the COVID-19 cruise tourism crisis, their leadership needs to look further and make contingency plans on how to prevent the next pandemic crisis and pending climate change crisis. Cruise line companies’ leadership should, therefore, ask themselves how the COVID-19 cruise tourism crisis did happen in the first place, and diligently work to change everything that went wrong. The COVID-19 pandemic has created a unique opportunity for cruise line companies to revise their corporate culture, reinvent their business models, enhance their human resource management, develop and embrace the risk and crisis management strategies, and adopt sustainable development. Based on the respondents’ answers, there are mixed opinions if there are positive ways how cruise line companies can get out of the COVID-19 cruise tourism crisis.

*We should not only look into the cruise companies and what they are doing. Have faith and hope. Talk to each other about what’s good. Somebody out there anywhere in the world is battling for their life in this pandemic. Come to think of the other perspective. After all of this darkness and rain, there will be light and rainbow and a brighter future. One day all of this will be the history which we will tell to our grandchildren. It would be a story of how we coped with a crisis and survived. All this will probably help us to be better people.*

(Cruise ship employee No. 6)

*To those people who are saying that we need to wait, put yourselves in our shoes, that is come on the ship that has a COVID-19 outbreak. This is day 61 of our isolation excluding the days when we were at sea before we started isolation. Almost 2.5 months without contact with other people. We are only asking for one thing, send us back home to our families.*

(Cruise ship employee No. 4)

The COVID-19 pandemic has exposed the unsustainable business model of cruise line companies. Although under normal circumstances working conditions onboard do not dehumanize cruise ship employees, the COVID-19 pandemic has certainly shown that for cruise line companies and the CDC, cruise ship employees do not matter in some profound way. The aforementioned condition is rooted in a systemic failure of cruise line companies’ leadership to understand what is happening with the COVID-19 pandemic, aggravated by the capitalism of the neoliberal era. Thus, as cruise line companies claim they are doing their best for the crew members’ repatriation, there is a dividing perception among cruise ship employees in regards to positive ways how cruise line companies can get out of the COVID-19 cruise tourism crisis. While some cruise ship employees see the light at the end of tunnel as the CDC’s No Sail Order [2] expires on 30 September 2020, others think that the long-awaited 30 September 2020 and the light at the end of the tunnel is nothing more than what Žižek [80] (pp. xi–xii) describes as: “probably the headlight of another train approaching from the opposite direction”.

On 3 February 2020, Princess Cruises confirmed that 10 people on the ship *Diamond Princess* had tested positive for COVID-19 [81] and on 11 March 2020, the World Health Organization officially declared COVID-19 a pandemic. At that time, there were 121,564 people infected, 4373 dead and 66,239 recovered including nearly 800 people aboard four cruise ships [82]. Shortly after that on 14 March 2020, members of the CLIA voluntarily suspended their cruise ship operations, followed by the CDC’s No Sail Order issued on 12 April 2020, which suspended all cruise operations until 30 September 2020 [2]. Lastly, on 17 May 2020, there were around 100,000 cruise ship employees stuck at sea without any certainty in regards to their repatriation to their homes [45]. All respondents but one were quite skeptical about the leadership of cruise line companies and their energetical pursuit to do everything in their power to get them home.

*No, I don’t think that office people are doing everything in their power to get us home. Crew members should be respected way more than they have been. If the company continues this horrible management of their crew members, the day will come when no one will work for them and they will fail as a company. Everything starts at the top. Horrible leadership! I have lost all faith in this company. I am just tired of all the lies and their failure to care about people. True colors came out in tough times. This is no way to treat anyone. So sad!*

(Cruise ship employee No. 8)

While being stuck at sea, cruise ship employees’ doubts in regards to cruise line companies’ leadership capabilities and their sincere efforts in pursuing crew members’ repatriation are boiling up. Thus, as despair, anxiety, depression and stress accumulate across various cruise ships, it appears that trust in cruise line companies’ leadership is diminishing, and as Walker [83] points out, some crew members are organizing protests, while others go even further by performing a hunger strike [84]. 

Thus, while cruise line companies’ leadership is battling the COVID-19 cruise tourism crisis, and they are looking into ways to tap into liquidity so they can stay afloat and avoid bankruptcy, they must not neglect their obligations outlined in The Maritime Labour Convention 2006 [44] “seafarers’ bill of rights”. All participants expressed an opinion that even when cruise line companies get bad publicity, they hope that they will find a way to solve the COVID-19 cruise tourism crisis

*Yes, they are doing their best. I don’t understand why media is bad mouthing cruise lines and they can’t understand that some countries are not accepting their crew members coming from cruise ships. Beside me, my sister is working on a cruise ship and we are from Trinidad and Tobago, and my sister’s boyfriend who also works on a cruise ship is from Nicaragua. All of us are not allowed by our countries to come back home. However, all you read is how cruise companies are bad and no one is accusing countries like ours who are not allowing us to come back home. This is hypocrisy! I can tell you that our company is looking after us while we are on board.*

(Cruise ship employee No. 9)

Crew ship employees believe that sometimes they are being used by media who are attracted to stories related to the COVID-19 pandemic and cruise tourism since, as Pooley [85] said, “if it bleeds, it leads.” Nevertheless, the COVID-19 pandemic should awaken cruise line companies’ leadership to the realization that exploitation of crew ship employees should be abandoned and replaced by social bonds between cruise line companies’ leadership and cruise ship employees. The bad publicity of the cruise line companies’ leadership during theCOVID-19 pandemic is a direct result of poor leadership skills, poor human resource management, nonexistent contingency plans, nonexistent crisis management, and nonexistent crisis communication strategies. Lastly, as cruise line companies’ leadership are delaying crucial decisions and continue to weigh the costs of crew members’ repatriation using charter flights and/or cruise ships while meeting the CDC’s No Sail Order [2], they are putting hardship on their crew ship employees.

## 5. Conclusions

Onboard working conditions under normal circumstances do not dehumanize cruise ship employees, however, the COVID-19 pandemic has managed to erase the feeling of joy from cruise ship employees while exposing the weakness of cruise line companies such as poor human resource management strategies, nonexistent contingency plans, and nonexistent crisis management. Accordingly, this study attempted to answer the question: What are the psychological effects of the COVID-19 pandemic on cruise ship employees stuck at sea?

### 5.1. COVID-19 Pandemic Psychological Effects on Cruise Ship Employees

The results of this study revealed that cruise line companies have poor human resource management strategies and that they did not have a contingency plan to manage this health and epidemiological type of crisis. Moreover, cruise line companies do not have a strategy for managing various negative psychological effects of the COVID-19 pandemic on cruise ship employees who are stuck at sea. Thus, since cruise line companies have to develop a comprehensive contingency plan for managing onboard COVID-19 outbreaks as a mandatory requirement set by the CDC’s No Sail Order [2], cruise line companies have to look further and develop strategies for managing anxiety, depression and stress of cruise ship employees during a pandemic and/or crisis. Cruise line companies need to embrace the philosophy defined by Mitroff [86] as “thinking about the unthinkable”.

Regarding the worries, fears and sleep disturbances experienced by cruise ship employees stuck at sea, it appears that they are related to fears of not being able to see their family and friends, not being able to provide financial support to their families or significant other and the feeling that they have lost control of their lives. Anxiety within cruise ship employees stuck at sea was inflated due to the fear of an uncertain future and economic recession. These findings are in line with Shigemura et al. [87] who argue that during a pandemic, worries and fears surge the anxiety levels in particularly healthy persons, and boost the manifestations of those with pre-existing mental disorders. As days become weeks and weeks become months, cruise ship employees experience sleep disturbances. The sleep disturbances of cruise ship employees stuck at sea were related to the combination of worries, fears, and anxiety, which ultimately affected their sleep quality. This finding is in line with Alvaro et al. [88] who in their systematic review on sleep disturbances, anxiety, and depression point to causality between anxiety and sleep quality due to a specific condition where anxious individuals experience difficulties to fall asleep and they wake up frequently during their sleep.

During the COVID-19 pandemic, cruise ship employees experience a lack of onboard social cohesion and lack of family and friends’ social support, which leads to the development of depression. Without an opportunity to socialize with fellow crew members due to restrictive social distancing, each cruise ship employee was left alone to face their worries and fears of uncertainty. Fear is an adaptive defense mechanism essential for survival with several biochemical processes as a response to potential threats [89]. Moreover, despair, a decline in well-being, combined with a loss of hope that the day of final repatriation is in sight has seriously affected how cruise ship employees feel, think, and act, causing the development of depression. These findings are supported by Garcia [90] who argues how chronic or disproportionate fear can harm individuals’ mental health and as it progresses it can lead to the development of various psychiatric disorders. Hence, as melancholy sweeps over cruise ship employees, their silent screams of existential despair go unheard by cruise line companies, the CDC, airlines, and national governments. 

Uncertainty of what the future holds, distress, and neurosis coupled with not being able to cope with the COVID-19 pandemic were the main components associated with the perceived stress of cruise ship employees. Unbearable uncertainty of what the future holds paralyzes cruise ship employees as they are preoccupied with the day of their final repatriation and loss of their only source of income. Peculiar conditions of cruise ship employees who are stuck at sea leave them with limited, if any strategies to manage their negative emotions. Thus emotion regulation strategies described by Diefendorff et al. [91] such as: (a) connecting with others so one could feel good; (b) working or keeping oneself busy; (c) enjoying pleasurable activities to improve one’s mood; (d) doing one’s best to solve a problem; are not applicable to cruise ship employees who are stuck on the sea due to the CDC’s No Sail Order [2]. Hence, cruise ship employees speak in an almost passive participant voice about their relentless complaints against diminished control of their own life. These findings are in line with Stein [92] who points out how the uncertainty of what the future holds, coupled with distress, and an inability to cope with the COVID-19 pandemic creates worries and anxiety among many people, leaving them with nothing but dread and despair.

This study showed that a state of hope and sense of belonging of cruise ship employees is hitting an all-time low. Cruise line companies’ leadership failed on multiple levels of human resource management, as they reached the point of being perceived by cruise ship employees as inauthentic and untrustworthy. Moreover, cruise line companies’ leaderships’ poor crisis communication strategies left the cruise ship employees almost without any hope in regards to their repatriation. The COVID-19 pandemic demonstrated that for cruise line companies and the CDC, cruise ship employees do not matter in some profound way. Even though it appears that hope has abandoned the cruise ship employees, there is a glimmer of a sense of belonging as cruise ship employees are willing to defend the image of cruise line companies under the ruthless judgment of mainstream media. Hence, as crises create opportunities, the COVID-19 pandemic has provided a unique opportunity for cruise line companies to revise their corporate culture and enhance their human resource management strategies. 

### 5.2. Practical Implications

The COVID-19 pandemic demonstrated that it is of paramount importance that cruise line companies create a comprehensive strategy in assisting their employees who are experiencing anxiety disorder and depression. In the case of a pandemic and/or crisis, cruise ship companies need to employ onboard psychologists who could assist employees with anxiety disorder and depression. The anxiety of cruise ship employees could be solved by Fairburn’s [93] cognitive behavior therapy. Cognitive behavioral therapy (CBT) is a well-established psychological treatment with robust effectiveness in treating depression and anxiety disorders [94]. Onboard leaders have to be approachable and they have to recognize crew members who are experiencing anxiety and depression. During the open conversation with cruise ship employees, onboard leaders have to be authentic and emphatic as they listen to worries, fears, and troubles of their crew members. It is a duty of onboard leaders to explain to cruise ship employees that it is normal to feel worried and anxious as uncertainty and loss of control are two key factors associated with stress and anxiety. Additionally, onboard leaders have to be supported by shore-side cruise line companies’ leaders with appropriate video content and digital CBT. In their study on the mental health burden of COVID-19, Da Silva Lopes and Jaspal [95] concluded that digital CBT can address all aspects of stress management and the management of worry and fear. Furthermore, Murphy et al. [96] suggest that delivering enhanced CBT remotely by video-calls delivers strong results in treating anxiety disorder. Practical aspects of CBT that onboard leaders can perform every day are: engaging crew members in the novel ways of protection against COVID-19; showing crew members reasons and positive ways to overcome despair; arranging outdoor activities that do not violate the CDC’s No Sail Order [2] (e.g., walks on the open decks, yoga and breathing classes with prescribed social distance); and engaging crew members in solving problems such as contacting their embassies for potential charter flights and final repatriation and sharing the information with shore-side leaders related to individual countries lockdown measures. 

Cruise line companies need to be transparent in their communication with cruise ship employees by providing them with accurate information in a timely manner. Cruise ship employees understand that during the COVID-19 cruise tourism crisis, cruise line companies’ leadership is struggling to keep companies afloat, while at the same time preventing potential takeovers by protecting the stock value. However, lack of information and/or inaccurate information can only boost the crisis. The COVID-19 pandemic demonstrated poor crisis communication of cruise line companies, since the main source of information and loudest spokespeople during the COVID-19 cruise tourism crisis was social media and mainstream media. It appears that cruise line companies neglect the fundamentals of crisis communication strategies, which is, as per Fink [97], managing the perception of reality by framing the public opinion. Although some cruise line companies created the slogan (e.g., “We will be back”), unfortunately, the slogan failed to capture a feeling of security so that cruise ship employees’ reaction was cold. Onboard leadership needs to understand who their crew members are and what they want to hear. In particular, cruise line employees are interested in: (a) their repatriation home; (b) financial support while they are away from home; and (c) their employment status. Thus, onboard leadership must communicate to them such information with empathy and compassion.

The cruise industry will certainly experience an exponential drop in employment due to the COVID-19 pandemic; however, this issue of unemployment will affect cruise ship employees’ family members as well. While being stuck at sea, cruise ship employees who are not getting paid cannot engage in activities such as searching for another job, looking into ways to increase their visibility on the labor market, rearranging their family finances by decreasing expenses, and outsourcing alternative resource for existential purposes. To soften the negative impact on cruise workers, cruise ship companies should provide a minimum basic salary for at least 3 months to all cruise ship employees who were affected by the COVID-19 pandemic, as well as support laid-off cruise ship employees in finding another job or allowing them to return to work if cruise line companies resume their operation. 

### 5.3. Limitations and Future Research

Due to its qualitative nature, this study cannot be generalized. It would be interesting to conduct similar research with additional online synchronous focus groups over time, to elicit in-depth information evolving during the course of the COVID-19 pandemic. The second limitation is the cross-sectional time horizon utilized in this study; thus, there is space for potential causality and reciprocal relationships among components [98]. Future studies should use a longitudinal time horizon to understand the complexities of the psychological effects of the COVID-19 pandemic on cruise ship employees stuck at sea. Moreover, a quantitative follow up study that is built on findings from this study would help improve our understanding of the psycholotablgical effects of the COVID-19 pandemic on cruise ship employees stuck at sea. Lastly, future studies can address the shortcomings of this study to gain a deeper understanding of the psychological effects of the COVID-19 pandemic on cruise ship employees stuck at sea. 

## Figures and Tables

**Table 1:** Demographic profile of participants.

Cruise Ship Code Number	Age	Nationality	Gender	Department/Sub Department/Working Role
employee No. 1	32	Croatia	Male	Hotel/Food and beverage/ Assistant waiter
employee No. 2	33	India	Male	Hotel/Housekeeping/ Assistant stateroom host
employee No. 3	29	Indonesia	Female	Hotel/Food and beverage/ Assistant bar server
employee No. 4	37	Serbia	Male	Hotel/Food and beverage/ Head waiter
employee No. 5	40	Philippines	Female	Hotel/Housekeeping /Stateroom host
employee No. 6	43	Philippines	Female	Hotel/Housekeeping/ Stateroom host
employee No. 7	26	UK	Female	Entertainment/Theater/ Main stage performer
employee No. 8	37	Philippines	Male	Marine and technical/Deck/ Garbage handler
employee No. 9	32	Trinidad and Tobago	Female	Hotel/Guest services/Purser
